# Matrix Topographical Cue-Mediated Myogenic Differentiation of Human Embryonic Stem Cell Derivatives

**DOI:** 10.3390/polym9110580

**Published:** 2017-11-05

**Authors:** Yongsung Hwang, Timothy Seo, Sara Hariri, Chulmin Choi, Shyni Varghese

**Affiliations:** 1 Department of Bioengineering, University of California, San Diego, CA 92521, USA; yshwang0428@sch.ac.kr (Y.H.); ynseo89@gmail.com (T.S.); s1hariri@eng.ucsd.edu (S.H.); 2 Soonchunhyang Institute of Medi-bio Science (SIMS), Soonchunhyang University, Cheonan-si, Chungcheongnam-do 31151, Korea; 3 Department of Mechanical and Aerospace Engineering, University of California, San Diego, CA 92521, USA; chulmin.choi@gmail.com; 4 Department of Biomedical Engineering, Mechanical Engineering and Materials Science and Orthopaedic Surgery, Duke University, Durham, NC 27708, USA

**Keywords:** human embryonic stem cells, topographical cues, soft lithography, myogenesis, cellular alignment, multinucleated myotubes

## Abstract

Biomaterials varying in physical properties, chemical composition and biofunctionalities can be used as powerful tools to regulate skeletal muscle-specific cellular behaviors, including myogenic differentiation of progenitor cells. Biomaterials with defined topographical cues (e.g., patterned substrates) can mediate cellular alignment of progenitor cells and improve myogenic differentiation. In this study, we employed soft lithography techniques to create substrates with microtopographical cues and used these substrates to study the effect of matrix topographical cues on myogenic differentiation of human embryonic stem cell (hESC)-derived mesodermal progenitor cells expressing platelet-derived growth factor receptor alpha (PDGFRA). Our results show that the majority (>80%) of PDGFRA^+^ cells on micropatterned polydimethylsiloxane (PDMS) substrates were aligned along the direction of the microgrooves and underwent robust myogenic differentiation compared to those on non-patterned surfaces. Matrix topography-mediated alignment of the mononucleated cells promoted their fusion resulting in mainly (~86%–93%) multinucleated myotube formation. Furthermore, when implanted, the cells on the micropatterned substrates showed enhanced in vivo survival (>5–7 times) and engraftment (>4–6 times) in cardiotoxin-injured tibialis anterior (TA) muscles of NOD/SCID mice compared to cells cultured on corresponding non-patterned substrates.

## 1. Introduction

Human pluripotent stem cells (hPSCs), which include human embryonic stem cells (hESCs) and human induced pluripotent stem cells (hiPSCs), can differentiate into all cell types in the human body. Hence, hPSCs are an ideal cell source for treating various debilitating diseases and developing technological platforms recapitulating various attributes of healthy- and diseased-tissue phenotypes. Recent studies have investigated the potential of using hPSC-derived cells to treat muscle injuries and muscle wasting diseases [1,2,3]. Employing stem cells to treat impaired skeletal muscle tissue requires experimental strategies that promote myogenic differentiation of these cells. A number of approaches, ranging from genetic manipulation, mRNA transfection, a cocktail of growth factors, cytokines and small molecules, have been used to direct myogenic differentiation of hPSCs [4,5,6].

Mammalian skeletal muscle tissue has a highly organized structure that consists of multiple parallel bundles of muscle fibers that are formed by the fusion of mononucleated myoblasts into multinucleated myotubes [7,8]. Over the last decades, many studies have utilized biomaterials with skeletal muscle-specific biochemical, biomechanical and topographical cues to promote myogenic differentiation of progenitor and stem cells [9,10,11,12]. In addition to promoting in vitro myogenic commitment, biomaterials have been used to promote in vivo survival and engraftment of transplanted cells in skeletal muscle [13,14,15].

Biomaterials with periodic grooves that facilitate orientation and alignment of cells have been shown to promote myogenic commitment and fusion of myoblasts and stem cells [16,17]. Various surface patterning technologies such as soft lithography have been used to endow biomaterials with topographical features of varying sizes [18,19]. Similarly, electrospun fiber-based biomaterials have also been shown to promote myogenic differentiation of stem and progenitor cells [20,21]. Most of the studies investigating the effect of topographical cues on myogenic differentiation and myotube formation used either murine myoblasts such as C2C12 cells or pre-committed adult stem cells [22,23,24,25].

Previously, we have shown that the mesodermal progenitor cells (PDGFRA^+^ cell population) derived from hESCs exhibit myogenic differentiation potential [26,27]. In this study, we investigated whether the topographical cues of the biomaterial could promote myogenic differentiation of these hESC-derived PDGFRA^+^ cells. Our studies show that controlling the cellular alignment of the cells through the matrix topography-mediated cues not only promoted myogenic differentiation of hESC-derived cells but also improved their in vivo survival and engraftment upon transplantation into cardiotoxin-injured skeletal muscles.

## 2. Materials and Methods

### 2.1. Maintenance of Human Embryonic Stem Cells (hESCs)

The HUES9-OCT4-GFP reporter cell line was generated and cultured as described earlier [28]. Both HUES9 and HUES9-OCT4-GFP cells were grown on mitotically inactivated MEF (mouse embryonic fibroblasts) feeder cells in growth medium (KnockOut DMEM, Thermo Fisher Scientific, Waltham, MA, USA) supplemented with 10% KSR (knockout serum replacement), 10% human plasmanate (Talecris Biotherapeutics), 1% NEAA (non-essential amino acids), 1% penicillin/streptomycin, 1% Gluta-MAX and 55 μM 2-mercaptoethanol in the presence of basic fibroblast growth factor (bFGF) (30 ng/mL, added daily). The cells were enzymatically dissociated with Accutase (Millipore, Burlington, MA, USA) and routinely passaged.

### 2.2. Derivation of PDGFRA^+^ Mesodermal Progenitors from hESCs

The PDGFRA^+^ mesodermal progenitor cells were derived from undifferentiated hESCs as described previously [27]. Briefly, undifferentiated colonies of HUES9 cells were enzymatically dissociated into single cells by incubating them with Accutase for 5 min. Approximately, 1.0 × 10^6^ of dissociated cells were seeded into each wells of an ultra-low-attachment 6-well plate and cultured in suspension to form embryoid bodies (EBs) in a 37 °C/5% CO_2_ incubator (Thermo Fisher Scientific, Carlsbad, CA, USA) for 9 days. The cells were cultured in an induction medium (high-glucose DMEM with 5% fetal bovine serum (FBS), 2 mM l-glutamine (Thermo Fisher Scientific), 100 nM dexamethasone (Tocris, Minneapolis, MN, USA), 100 µM hydrocortisone (Sigma-Aldrich, St. Louis, MI, USA), 1% penicillin/streptomycin (Thermo Fisher Scientific), 10 µM transferrin (Thermo Fisher Scientific), 860.9 nM recombinant insulin (Thermo Fisher Scientific), 20 nM progesterone (Thermo Fisher Scientific), 100.1 µM putrescine (Thermo Fisher Scientific), and 30.1 nM selenite (Thermo Fisher Scientific) with the medium changing every other day. Next, the EBs in each well were transferred to a Matrigel-coated 10 cm dish (1:25 diluted in KnockOut DMEM; BD Biosciences, Franklin Lakes, NJ, USA) and continued to culture in the induction medium. The cells were cultured for another 7 days, which allowed a large number of cells to migrate out of the EBs. The cells that migrated out of the EBs were enzymatically detached with Accutase and filtered through a cell strainer having a pore size of 40 μm. The isolated single cells were enriched for PDGFRA^+^/OCT4-GFP^−^ single cells by fluorescence-activated cell sorting (FACS).

### 2.3. Fluorescence-Activated Cell Sorting (FACS) Analysis

To isolate PDGFRA^+^ mesodermal progenitor cells, the hESC-derived single cells were re-suspended in a buffer containing 2% FBS and 0.09% sodium azide in calcium-/magnesium-free phosphate-buffered saline (PBS) (BD Biosciences). The hESC-derived cells were stained with either Alexa Fluor 647-conjugated PDGFRA or Alexa Fluor 647-conjugated mouse IgM, ĸ isotype control antibodies (Biolegend) on ice for 30 min. The cells were then washed with fresh PBS for three times, resuspended in the buffer solution, and FACS-sorted for PDGFRA^+^ cells by using BD Biosystems’ FACSCanto. Data were analyzed with the CellQuest Pro software (BD Biosciences).

### 2.4. Fabrication of Micropatterned PDMS Substrates

PDMS substrates displaying line patterns of two different widths, 100 and 200 μm, were generated. Standard soft lithography technique was used to fabricate Si master molds, which were then used to generate the micropatterned PDMS substrates (Sylgard 184 silicone elastomer, Dow Corning, Midland, MI, USA) [29]. Briefly, Si wafers were spin-coated with SU8 2050 negative photoresist (PR) polymer (Microchem Corp, Newton, MA, USA) at 2500 rpm for 60 s to get a thickness of ~60 μm, followed by soft-baking at 95 °C for 9 min. To create microgrooved patterns (i.e., line patterns), the negative PR-coated Si wafer was exposed to UV at 331 W for 15 s under a photomask using Karl Suss MA6 Mask Aligner (SUSS Micro Tec SE, Garching, Germany). Subsequently, the Si wafer was hard-baked at 95 °C for 7 min, and micropatterned surfaces were developed in SU8 developer solution (Microchem Corp) for 5 min. Next, the solutions containing SYLGARD 184 Silicone Elastomer Curing Agent and Elastomer Base (Dow Corning Corporation, Midland, MI, USA) were mixed in 1:10 ratio, poured onto the Si master mold and degassed for approximately 30 min. Once all bubbles disappeared from the solution, Si mold with PDMS solution was placed in an 80 °C oven overnight. The micropatterned PDMS substrates were peeled off from the Si wafer, sterilized in ethanol for 30 min and washed with sterile PBS (2% penicillin/streptomycin) three times a day for a week. Non-patterned PDMS substrates were also fabricated and processed under identical conditions.

### 2.5. Surface Characterization by Scanning Electron Microscopy (SEM)

The micropatterned and non-patterned PDMS substrates were sputter-coated with Ir for 7 s and imaged using a scanning electron microscope (Philips XL30 ESEM, Amsterdam, the Netherlands).

### 2.6. Preparation of PDMS Substrates for Cell Culture

Prior to cell culture, the PDMS substrates were cut into circular discs with a diameter of 22 mm, sterilized in 70% ethanol and washed extensively with fresh PBS to wash off residual ethanol and other unreacted reactants. The sterilized circular PDMS discs (1 disc per well) were subsequently placed in 12-well tissue culture plates (TCPS). To promote initial cell adhesion, the PDMS substrates were coated with growth factor-reduced Matrigel (1:25 diluted in DMEM; BD Biosciences) as previously reported [30]. The hESC-derived PDGFRA^+^ cells were plated onto micropatterned and non-patterned PDMS substrates at an initial cell density of 1 × 10^4^ cells/cm^2^ and cultured in the induction medium. The induction medium was changed every other day throughout their culture.

### 2.7. Immunofluorescence Staining

Cells cultured on micropatterned (microgroove widths of 100 or 200 µm) or non-patterned PDMS substrates were stained against MF20 (1:200; Developmental Studies Hybridoma Bank, Iowa City, IA, USA) and Desmin (DES) (1:250; Abcam, Cambridge, UK). The cell-laden substrates were fixed with 4% paraformaldehyde (PFA) for 10 min at room temperature, permeabilized, and treated with a blocking solution containing 0.1% (*v*/*v*) Triton X-100 and 3% (*w*/*v*) bovine serum albumin (BSA) in PBS for 1 h. The samples were incubated with the aforementioned primary antibodies overnight at 4 °C. After washing with fresh PBS (~3 times), samples were incubated with the following secondary antibodies: goat anti-mouse Alexa 488 (1:250; Life Technologies, Carlsbad, CA, USA) or goat anti-rabbit Alexa 546 (1:250; Life Technologies) for 1 h at room temperature. To assess the morphology and alignment of cells cultured on micropatterned or non-patterned PDMS substrates, the samples were incubated with phalloidin Alexa-Fluor 488 (1:100; Life Technologies) for 1 h at room temperature. To stain nuclei, Hoechst 33342 (2 μg/mL; Life Technologies) was used. For immunofluorescence staining of tibialis anterior (TA) muscles transplanted with donor cells, samples were first embedded in optimal temperature cutting compound (OCT) for cryosectioning. The sections (~15 µm thickness) were fixed with 4% PFA for 10 min at room temperature, permeabilized with 0.3% Triton X-100, blocked with 3% BSA for 1 h at room temperature, and stained with anti-human lamin A/C (1:100; Vector Laboratories, Burlingame, CA, USA) and rabbit anti-laminin (1:200; Abcam) to visualize transplanted donor cell survival and their engraftment within the host tissue. All images were acquired using a fluorescence microscope (Carl Zeiss; Axio Observer A1, Thornwood, NY, USA).

### 2.8. Image Analysis

Two-dimensional fast Fourier transform (FFT) image processing was used to characterize the differences in substrate topography-mediated cellular alignments. NIH ImageJ software was used to convert raw images of F-actin staining into the 8-bit greyscale TIFF formats and these images were cropped into 1024 by 1024 pixels. The cropped images were then converted to the frequency domain by FFT transformation, rotated by 90 degrees and analyzed by an oval profile plug-in to conduct a radial summation of the pixel intensities from 0 to 360°. Cell nuclei alignment and orientation of the cells cultured on micropatterned and non-patterned PDMS substrates were determined by NIH ImageJ software [31]. The alignment of each nucleus was quantified based on the orientation of the major elliptical axis of each nucleus with respect to the horizontal axis. These angles of each nucleus were normalized to the mean orientation angle of all nuclei, and the frequency of alignment angles was binned in 10 degree increments to their mean orientation. The nuclear shape index was quantified as aspect ratio, which is defined as the ratio of major axis to minor axis of each nucleus, to evaluate the elongation of nuclei. To quantify the extent of myogenic differentiation of hESC-derived PDGFRA^+^ cells, differentiation and fusion indices were defined. The fraction of MF20-positive cells to the total number of cells was defined as differentiation index [32]. Similarly, the fraction of multinucleated myotubes containing 3 or more nuclei to the total number of MF20-positive cells was presented as the fusion index.

### 2.9. RNA Isolation and Quantitative PCR

Total RNA was extracted from three biological replicates with TRIzol (Invitrogen, Carlsbad, USA) and reverse transcribed using iScript cDNA synthesis kit (Bio-Rad, Marnes-la-Coquette, France), following the manufacturer’s instructions. Quantitative polymerase chain reaction (qPCR) was performed by using SYBR Select Master Mix (Life Technologies) and ABI Prism 7300 Sequence Detection System (Applied Biosystems, Foster City, CA, USA) [33]. The expression levels of target genes were normalized to glyceraldehyde 3-phosphate dehydrogenase (GAPDH) expression as reference and delta Ct values were calculated as *C*t_target_ − *C*t_reference_, and the relative fold inductions were presented as 2^−∆∆*C*t^. The primers used in this study are listed in Supplementary Table S1 (Supplementary Materials).

### 2.10. Cell Transplantation in NOD/SCID Cardiotoxin Injury Model

Animal experiments were performed according to the protocols approved by Institutional Animal Care and Use Committee (IACUC) of the University of California, San Diego (Protocol number: S07411). Twenty-four hours prior to cell transplantation, TA muscles of 2-month-old immune-deficient NOD.CB17-Prkdc^scid^/J (hereafter, NOD/SCID) mice were injured by intramuscular injection of 20 μL cardiotoxin (10μM; Sigma, cat. # C9759, Mendota Heights, MN, USA). To prepare animals for cell transplantation, the NOD/SCID mice were anesthetized by using ketamine (100 mg·kg^−1^) and xylazine (10 mg·kg^−1^). Approximately 5 × 10^5^ PDGFRA^+^ cells from micropatterned (100 and 200 μm) and non-patterned substrates cultured for 21 days in induction medium were collected and suspended in 20 μL of PBS and injected into the TA muscles. Two weeks post-transplantation, the TA muscles were harvested and embedded in OCT for cryosectioning. Survival and engraftment of the transplanted donor cells were histologically evaluated. Quantification of histological sections for viable donor cells was performed by counting the number of lamin A/C positive nuclei. Three serial sections from each muscle sample (no pattern, 100 µm, and 200 µm) were analyzed for each biological triplicate. Engraftment is defined as any cell that is fused with host myofibers or located underneath the basal lamina. To quantify engraftment, number of viable donor cells centrally located within a myofiber was counted and presented as the percentage of engraftment.

### 2.11. Statistical Analysis

All values are presented as mean ± standard deviation and statistical significance was determined by two-tailed unpaired Student’s t-test or single-factor analysis of variance (ANOVA) with Tukey’s multiple comparison test (* *p* < 0.05, ** *p* < 0.01 and *** *p* < 0.001). GraphPad Prism (GraphPad Software, La Jolla, CA, USA) was used to perform all the statistical analysis.

## 3. Results and Discussion

### 3.1. Substrate Topographical Cue-Mediated Actin Cytoskeletal Organization and Cellular Alignment

**Figure 1:**
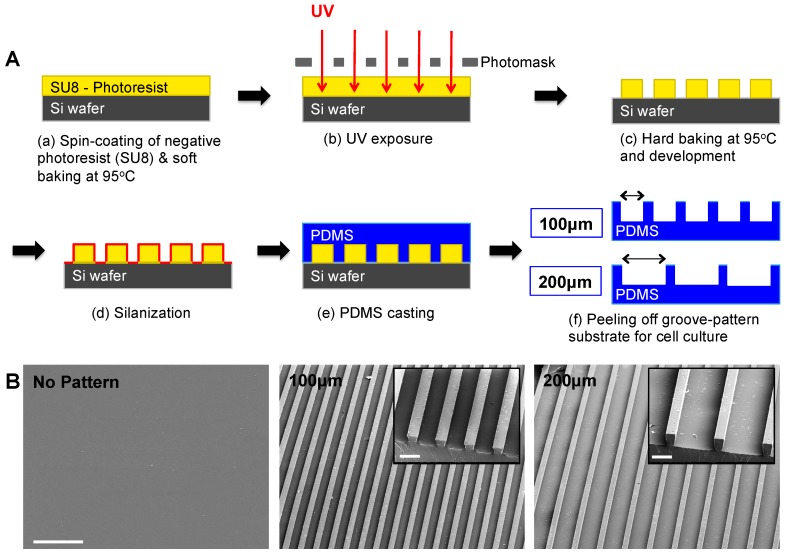
Fabrication and characterization of micropatterned PDMS cell culture substrates. (**A**) Schematic illustration of standard soft lithography procedures for fabrication of micropatterned PDMS cell culture substrates. (**B**) Surface characterization of various PDMS cell culture substrates by SEM. SEM images show non-patterned (left) and micropatterned substrates (100 μm grooves—middle and 200 μm grooves—right). Corresponding higher-magnification SEM images are also presented in the insets. Scale bars = 500 μm and 100 μm (inset).

Since topographical cues have been shown to regulate various functions of myoblasts [31], we examined the effect of substrate topography on myogenic differentiation of hESC-derived PDGRA^+^ cells by using micropatterned PDMS substrates with varying groove widths (100 or 200 µm) while exhibiting the same height of 60 µm. A schematic illustration of the PDMS substrate fabrication is shown in Figure 1A. The topographical features of the substrates were examined by SEM and their representative images are shown in Figure 1B.

**Figure 2:**
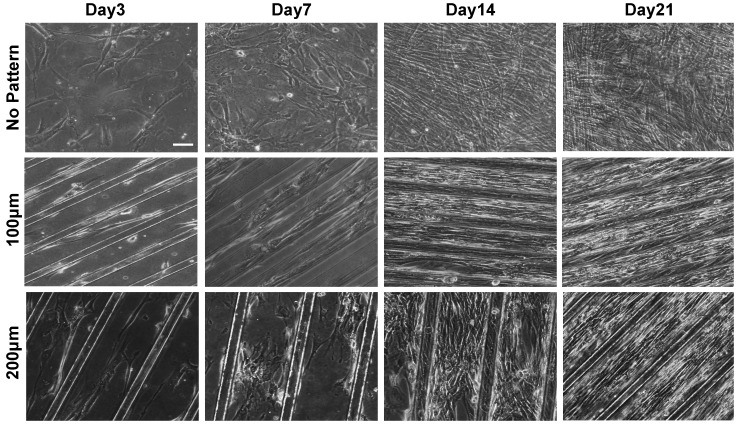
Characterization of cellular morphology of hESC-derived PDGFRA^+^ myogenic progenitor cells cultured on PDMS cell culture substrates with various topographical features. Phase contrast images of PDGFRA^+^ myogenic progenitor cells undergoing myogenic differentiation at different time points (top row—non-patterned substrate; middle row—micropatterned substrates having 100 μm grooves; bottom row—micropatterned substrates having 200 μm grooves). Scale bar = 100 μm.

The morphological changes of the cells responding to the topographical features of PDMS substrates were examined as a function of time, initially, by using phase contrast microscopy. As shown in Figure 2, the cells adhered onto the Matrigel-coated PDMS substrates and exhibited spindle-like shape within 48 h of in vitro culture, regardless of the topographical features of the substrate. However, with culture time, the cells on the micropatterned substrates were elongated and aligned along the orientation of the microgroove. Amongst the micropatterned surfaces, cells cultured on substrates with a groove width of 100 µm were found to exhibit a higher degree of elongation and alignment as compared to that on 200 µm groove width. The matrix topography-mediated alignment of cells was evident as early as day three of culture. On the contrary, cells on non-patterned PDMS showed random organization.

**Figure 3:**
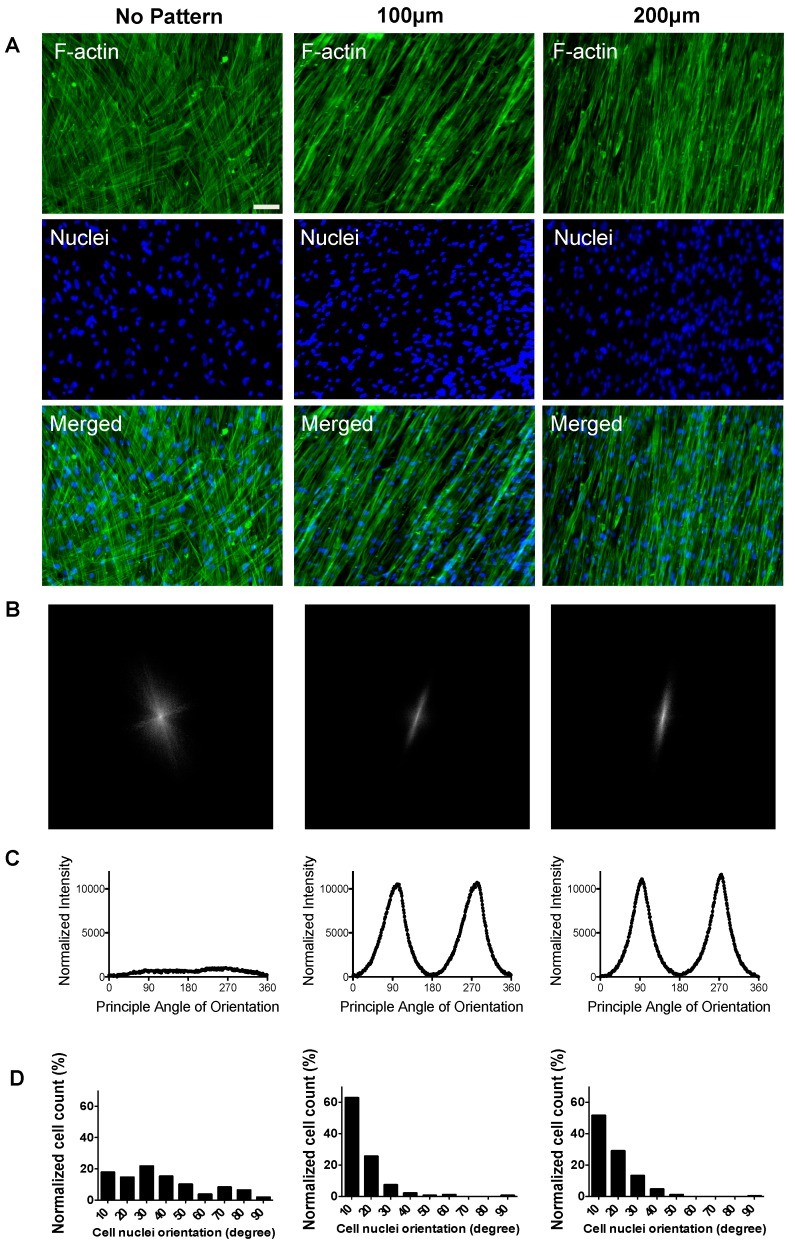
Topological cue-guided cellular alignment of hESC-derived PDGFRA^+^ myogenic progenitor cells. (**A**) F-actin immunofluorescence staining of hESC-derived myogenic progenitor cells cultured on PDMS substrates with various topographical features for 21 days (left column—non-patterned substrate; middle column—micropatterned substrate having 100 μm grooves; right column—micropatterned substrate having 200 μm grooves). Scale bar = 100 μm. (**B**) Frequency domain plots analyzed by the 2D FFT transformation. (**C**) FFT alignment histogram generated by a radial summation of the pixel intensities from 0 to 360°. (**D**) Cell nuclei orientation of hESC-derived myogenic progenitor cells cultured on PDMS cell culture substrates with various topographical features.

Since actin cytoskeletal organization is known to be closely involved in myogenic differentiation and fusion of myoblasts [34], the topographical cue-mediated actin cytoskeletal organization of the cells was also examined. As shown in Figure 3A, F-actin staining of the cells cultured on non-patterned PDMS showed a random orientation and lack of alignment (first column in Figure 3A). On the other hand, cells on the micropatterned PDMS substrates showed a parallel, unidirectional organization of actin along the direction of microgrooves with an actin-rich cytoplasmic polarization (second and third columns in Figure 3A).

Substrate topographical cue-mediated cellular alignment was further assessed by 2D fast Fourier transform (FFT) analysis on the F-actin staining images of the cells (Figure 3A). The F-actin images were converted into mathematically defined FFT frequency plots (Figure 3B), containing grayscale pixels that were distributed in patterns around the origin. This can be used to depict the degree of cellular alignment, where highly dispersed data points around the center of the FFT plot indicate random cell orientation. As seen in Figure 3B, the frequency plot for the cells on non-patterned substrates showed a symmetrical projection about the origin of plot (Figure 3B, left). Their corresponding FFT alignment histogram shows no distinctive peaks at any angle between 0–360° (Figure 3C, left). These randomly distributed pixel intensity patterns suggest random organization of the cells on non-patterned PDMS substrates. In contrast, the FFT frequency plots of cells cultured on the micropatterned PDMS substrates showed values concentrated along a line angled at ~70 degrees from the horizontal axis of the plot (Figure 3B, center and right). In addition, their corresponding FFT alignment histograms demonstrate two distinctive peaks, indicating a higher degree of alignment of cells cultured on micropatterned substrates (Figure 3C, center and right).

Apart from the actin cytoskeleton organization and alignment, we also analyzed topography-mediated alignment of cell nuclei, the ratio of the major axis of nuclei to the minor axis of nuclei. Degree of nuclei elongation has previously been shown to affect in vitro myoblast function [35]. As shown in Figure 3D, cells cultured on micropatterned PDMS substrates displayed higher nuclei alignment as compared to non-patterned substrates. Approximately 88.3% (+/−20° to the mean orientation angle) of cells cultured on 100 µm microgrooves showed alignment to the mean orientation angle as compared to 80.7% of cells on 200 µm microgrooves and 32.4% of cells cultured on non-patterned substrates. The results clearly suggest that cells cultured on non-patterned substrates are randomly oriented with a very small degree of nuclei alignment.

### 3.2. Effect of Matrix Topographical Cue-Mediated Cellular Alignment on In Vitro Myogenesis of hESC-Derived PDGFRA^+^ Cells

**Figure 4:**
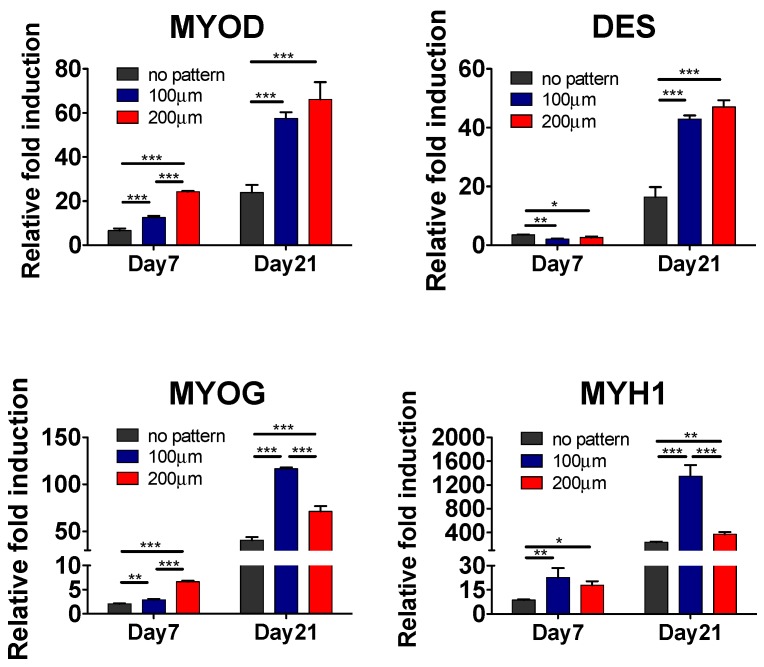
In vitro myogenic differentiation of hESC-derived PDGFRA^+^ myogenic progenitor cells cultured on PDMS substrates with various topographical features. Gene expression profiles of PDGFRA^+^ cells cultured on PDMS cell culture substrates with various topographical features. Statistical analysis was performed among cells cultured in different cell culture substrates within the same time point. * *p* < 0.05, ** *p* < 0.01 and *** *p* < 0.001.

**Figure 5:**
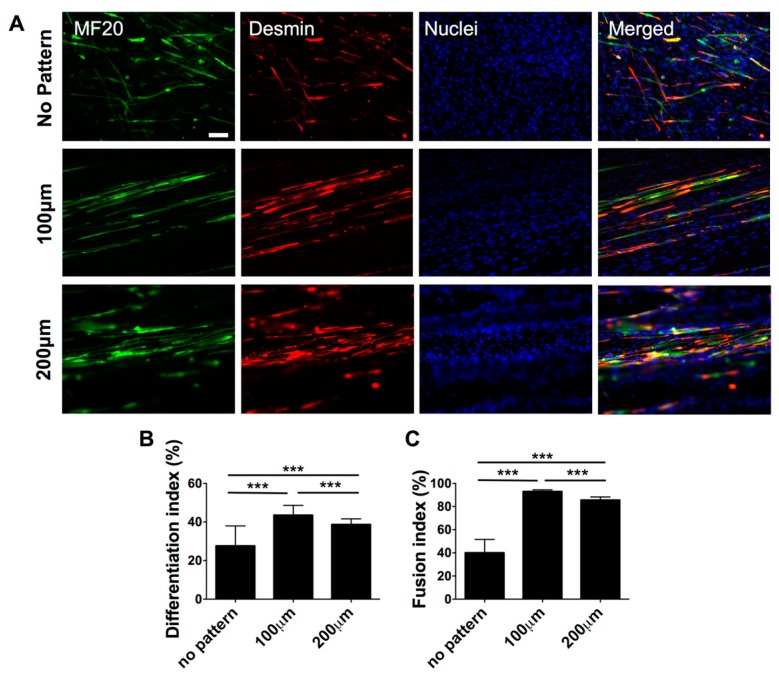
Terminal myogenic differentiation of hESC-derived PDGFRA^+^ myogenic progenitor cells cultured on PDMS cell culture substrates with various topographical features characterized by immunofluorescence staining and cell shape analyses. (**A**) Immunofluorescence staining for MF20 (green) and Desmin (red) of PDGFRA^+^ cells cultured on PDMS cell culture substrates with various topographical features. Scale bar = 100 μm. (**B**) Estimated differentiation indices of hESC-derived myogenic progenitor cells. (**C**) Estimated fusion indices of differentiated cells (MF20^+^ cells) cultured on PDMS substrates with various topographical features (no pattern, 100, and 200 µm). *n* = 209, 325 and 258, respectively. *** *p* < 0.001.

Having demonstrated the substrate topography-mediated cytoskeletal organization, we next examined whether this cellular alignment could promote myogenic commitment of hESC-derived PDGFRA^+^ cells. The gene expression profile, as shown in Figure 4, revealed that the cells cultured on micropatterned PDMS substrates exhibited a significantly higher expression of various myogenic markers, such as *MYOD*, *DES*, *MYOG* and *MYH1*, compared to their non-patterned counterparts. Moreover, cells cultured on the micropatterned substrates with a groove width of 100 µm showed higher upregulation of late myogenic markers, *MYOG* and *MYH1*, after 21 days of in vitro culture. The gene expression profile was further corroborated by the immunofluorescence staining for sarcomeric myosin (MF20) and Desmin, which are typically found in differentiated myoblasts (Figure 5A).

To quantify the extent of myogenic differentiation, we have calculated the differentiation index, defined as the fraction of MF20^+^ cells from the total number of cells. The differentiation index showed a significantly higher number of differentiated cells on micropatterned substrates (both 100 and 200 µm) compared to non-patterned substrates (Figure 5B). In addition to the differentiation index, we also evaluated the fraction of MF20^+^ multinucleated myotubes having three or more nuclei, termed as fusion index. Akin to the differentiation index, a significant fraction of cells cultured on micropatterned substrates were found to be multinucleated compared to cells cultured on non-patterned substrates. Amongst the micropatterned substrates, cells cultured on substrates with a groove width of 100 µm showed the highest differentiation and fusion indices. Together, the data from gene expression analysis as well as the immunohistochemistry confirm that matrix topography-mediated cues mediated changes in actin cytoskeleton structures of hESC-derived cells, can promote their myogenesis and facilitate fusion to form multinucleated myotubes.

### 3.3. In Vivo Engraftment of hESC-Derived Myogenic Progenitors

**Figure 6:**
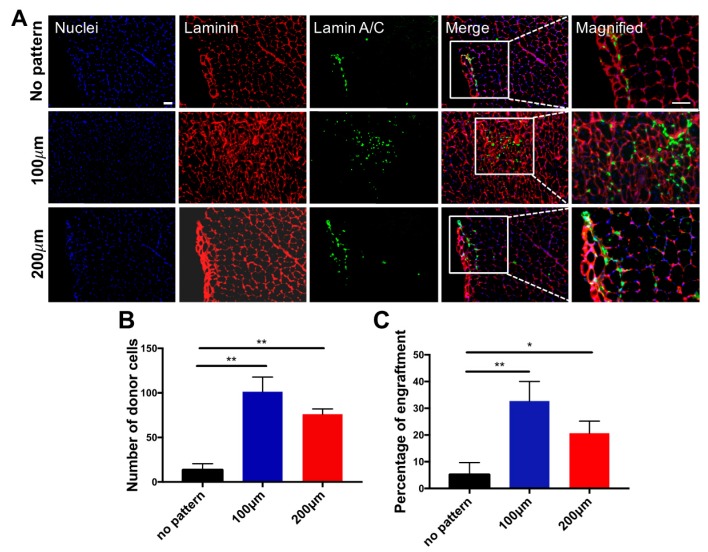
Engraftment of myogenic progenitor cells in cardiotoxin-injured mice. (**A**) Immunofluorescence staining of TA muscle of NOD/SCID mice transplanted with cells cultured on non-patterned substrates (top row), 100 μm-width grooves (middle row) and 200 μm-width grooves (bottom row) for 21 days of in vitro differentiation prior to the transplantation. Muscle sections were stained for mouse laminin (red), anti-human lamin A/C (green) and nuclei (blue). Scale bar = 50 μm; (**B**) Estimated number of total donor nuclei; (**C**) Estimated percentage of fused donor cells 14 days post-transplantation. * *p* < 0.05 and ** *p* < 0.01.

We next determined whether the matrix topography-mediated in vitro pre-conditioning of the cells has any effect on their in vivo survival and engraftment upon cell transplantation. The donor cells cultured on micropatterned (100 and 200 µm) and non-patterned substrates were transplanted into cardiotoxin-injured TA muscles of 2-month-old immunodeficient NOD/SCID mice. Muscles treated with donor cells were characterized 14 days post-transplantation to assess the viability and contribution of the transplanted cells to the regeneration of the host tissues. Regardless of the topographical differences for pre-conditioning cells prior to cell transplantation, histological analyses of the host tissue demonstrated the presence of donor cells, as shown in Figure 6A. More importantly, a significantly higher number of viable donor cells were found when the transplanted cells were cultured on micropatterned substrates in vitro prior to transplantation (Figure 6B). Consistent with these findings, the number of donor cells fused with the host myofibers was also found to be significantly higher for the cells that were cultured on micropatterned substrates prior to their transplantation (Figure 6C). This could be attributed to the higher extent of myogenic commitment of the cells on micropatterned substrates, as determined by the gene expression profiles of the progenitor cells in vitro. Moreover, cells cultured on the micropatterned substrates showed fully matured expression levels of late myogenic markers, such as MYOG and MYH1, as compared to the cells derived on non-patterned substrates. Our in vivo results could implicate the importance of the extent of in vitro myogenic commitment of progenitor cells on in vivo survival and function of transplanted cells. These results are in accordance with our previous reports, which suggests a strong relationship between the extent of differentiation of the transplanted cells and their in vivo function [36]. For example, we demonstrated that pre-committed myogenic progenitor cells displaying later-stage myogenic markers and mesodermal progenitor cells primed with a recombinant WNT3A protein promote in vivo survival of donor cells and their engraftment within the host myofibers [13,26,27]. Furthermore, no evidence of teratoma formation in any of the mice transplanted with cells cultured on both non-patterned and micropatterned substrates was observed.

## 4. Conclusions

In conclusion, the results summarized in this study illustrate the roles of matrix topographical cues on the fusion and differentiation of mononucleated myogenic progenitors into matured multinucleated myotubes, and highlight the importance of the degree of in vitro myogenic commitment of donor cells toward the in vivo regenerative potential upon cell transplantation.

## Supplementary Materials

The following are available online at www.mdpi.com/2073-4360/9/11/580/s1. Supplementary Figure S1: Cell shape analysis, Table S1: List of primers used for quantitative PCR.
