# Supplementary material for: Matrix Topographical Cue-Mediated Myogenic Differentiation of Human Embryonic Stem Cell Derivatives
Source: Polymers (Basel). 2017 Nov 5;9(11):580. doi: 10.3390/polym9110580 (PMC6418725; doi:10.3390/polym9110580)
Supplement: Supplementary file 1 [file polymers-09-00580-s001.zip › Table S1.pdf]

Gene	Primer Sequence (5' to 3')
MYOD	F-AGC ACT ACA GCG GCG ACT C<br>R-TAG TAG GCG CCT TCG TAG CA
DES	F-GAA GCT GCT GGA GGG AGA G<br>R-ATG GAC CTC AGA ACC CCT TT
MYOG	F-CAG CTC CCT CAA CCA GGA G<br>R-GCT GTG AGA GCT GCA TTC G
MYH1	F-TCT TGG ACA TTG CTG GCT TT<br>R-TCC ACT CAA TGC CTT CCT TC
18s	F-CCC TGT AAT TGG AAT GAG TCC ACT T<br>R-ACG CTA TTG GAG CTG GAA TTA C
