# Supplementary figures and images for: Matrix Topographical Cue-Mediated Myogenic Differentiation of Human Embryonic Stem Cell Derivatives
Source: Polymers (Basel). 2017 Nov 5;9(11):580. doi: 10.3390/polym9110580 (PMC6418725; doi:10.3390/polym9110580)

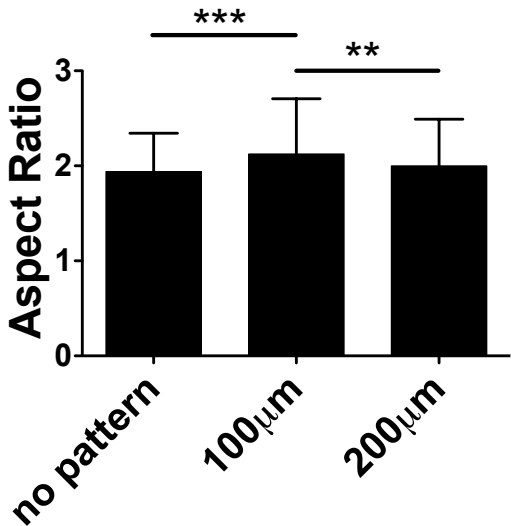

Supplement: Supplementary file 1 [file polymers-09-00580-s001.zip › Figure S1.pdf]
